# Methodological approach for assessing motor cortical excitability changes with single-pulse transcranial magnetic stimulation

**DOI:** 10.1016/j.mex.2023.102451

**Published:** 2023-10-18

**Authors:** Emmanuel Ortega-Robles, Jessica Cantillo-Negrete, Ruben I. Carino-Escobar, Oscar Arias-Carrión

**Affiliations:** a Unidad de Trastornos del Movimiento y Sueño, Hospital General Dr. Manuel Gea González, Mexico City 14080, Mexico; b División de Investigación en Neurociencias Clínica, Instituto Nacional de Rehabilitación Luis Guillermo Ibarra Ibarra, Mexico City 14389, Mexico

**Keywords:** Transcranial magnetic stimulation, Motor evoked potentials, Primary motor cortex, Cortical excitability, Evaluating changes in motor cortical excitability through single-pulse transcranial magnetic stimulation pre- and post-intervention

## Abstract

Transcranial Magnetic Stimulation (TMS) serves as a crucial tool in evaluating motor cortex excitability by applying short magnetic pulses to the skull, inducing neuron depolarization in the cerebral cortex through electromagnetic induction. This technique leads to the activation of specific skeletal muscles recorded as Motor-Evoked Potentials (MEPs) through electromyography. Although various methodologies assess cortical excitability with TMS, measuring MEP amplitudes offers a straightforward approach, especially when comparing excitability states pre- and post-interventions designed to alter cortical excitability. Despite TMS's widespread use, the absence of a standardized procedure for such measurements in existing literature hinders the comparison of results across different studies. This paper proposes a standardized procedure for assessing changes in motor cortical excitability using single-pulse TMS pre- and post-intervention. The recommended approach utilizes an intensity equating to half of the MEP's maximum amplitude, thereby ensuring equal likelihood of amplitude increase or decrease, providing a consistent basis for future studies and facilitating meaningful comparisons of results.

• A method for assessing changes in motor cortical excitability using single-pulse TMS before and after a specified intervention.
• We recommend using an intensity equal to half of the MEP's maximum amplitude during evaluations to objectively assess motor cortical excitability changes post-intervention.

## Graphical abstract

**Figure:**
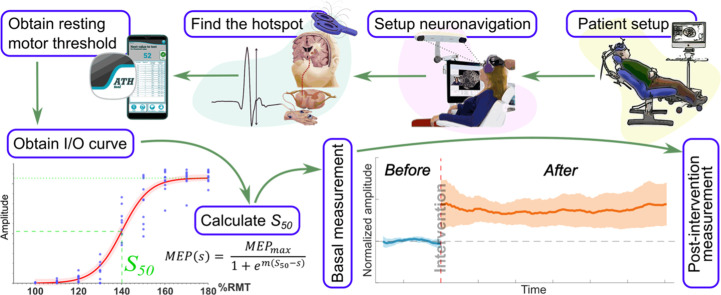

Specifications table

**Table:** 

Subject area:	Neuroscience
More specific subject area:	Transcranial Magnetic Stimulation
Name of your method:	Evaluating changes in motor cortical excitability through single-pulse transcranial magnetic stimulation pre- and post-intervention
Name and reference of original method:	None
Resource availability:	Does not apply

## Method details

### Transcranial magnetic stimulation

Transcranial Magnetic Stimulation (TMS) is a non-invasive, safe technique developed 38 years ago by Barker and colleagues [1]. Initially, TMS was employed to assess the corticospinal tract's integrity and has since evolved to stimulate various cerebral cortex regions and induce changes through neuromodulation. The technique involves generating a short-duration, high-intensity electromagnetic field by passing an electric current pulse through a coil. This magnetic field can penetrate the scalp and skull to induce neuron depolarization in the brain tissue through electromagnetic induction.

Different stimulation coils, such as circular, figure-of-eight, H-coils, and double and triple coils, have been designed for TMS, each providing different areas and depths of neural activation [2]. Stimulators can also deliver monophasic, half-wave, or biphasic pulses, thereby altering the waveform and direction of the induced current in the tissue [3]. The neuronal response to stimulation is complex, influenced by various factors, including stimulus duration, intensity, magnetic field orientation, induced current direction, neuronal morphology, and electrical properties [4]. Repetitive application of stimuli can either increase or decrease neuronal excitability, with effects being transient or long-lasting, as seen in Repetitive Transcranial Magnetic Stimulation (rTMS) neuromodulation [5]. TMS has become invaluable in both clinical practice and research due to its capability to obtain parameters related to cerebral cortex excitability (via single or paired-pulse stimulation) and modulate neuronal excitability (via rTMS).

In the clinical realm, TMS is instrumental in diagnosing upper motor neuron pathologies, distinguishing movement disorders, and assisting in the assessment and monitoring of various conditions, including dementia, migraine, epilepsy, and chronic pain [6,7]. Furthermore, rTMS serves as a therapeutic approach for managing psychiatric disorders [8] and plays a vital role in cerebral cortex mapping for preoperative purposes and evaluating motor, sensory, or cognitive functions [9]. Additionally, rTMS allows for the temporary activation or deactivation of specific cerebral cortex sites, creating “virtual lesions” that are invaluable for investigating functions and associations between multiple brain structures [10].

In research, it is crucial to measure neuronal states before and after interventions, such as pharmacological treatments, specific area activation or inhibition following an rTMS protocol, or tasks that activate particular cerebral cortex regions. While various techniques have been described for this purpose, this article focuses on outlining a straightforward method for implementing single-pulse TMS for motor cortex assessments.

## Methods for assessing motor cortex excitability

Cortical excitability is the ease with which neurons generate action potentials. This excitability can be quantified by evaluating the response strength of cortical neurons to a given stimulation. Various methods, TMS, Electroencephalography (EEG), Functional Magnetic Resonance Imaging (fMRI), and the combination of TMS with EEG (TMS+EEG), are employed for measuring cortical excitability [11], [12], [13]. However, TMS-evoked motor potentials provide a direct approach for examining motor cortex excitability specifically.

As previously noted, a TMS pulse applied to the primary motor cortex induces an electric field in brain tissue. Depending on this field's intensity, the stimulation can depolarize the axonal terminals of pyramidal cells and inhibitory interneurons in the cortex's layers II, III, and V. This action induces a discharge of synchronous action potentials that travel through the corticospinal tract to the spinal cord's ventral horn. Here, they activate lower motor neurons innervating specific muscles, generating a Motor-Evoked Potential (MEP) easily recorded using Electromyography (EMG) [4,14].

The motor cortex is frequently studied using TMS due to the ease of objectively measuring its excitability through skeletal muscle electromyography. This practice has led to a significant body of knowledge about TMS's effects on the cerebral cortex through studies in this area. Assessing the motor cortex's excitability is crucial, even for non-motor function studies, as many safety and stimulation parameters applied to other cortical areas are based on motor cortex excitability findings [15].

Various techniques have been developed to assess motor cortex excitability parameters using TMS. These include measuring motor threshold, MEP amplitude, the input/output curve, and conducting paired-pulse measurements.

## Motor threshold

The Motor Threshold (MT) denotes the stimulation intensity needed to reliably generate MEPs. It is conventionally defined as the minimum intensity eliciting MEPs in 10 out of 20 trials with a peak-to-peak amplitude of ≥50 µV [16]. MT offers insight into the initiation of action potentials by cortical neurons, indicating their average hyperpolarization or depolarization level.

MT is vital primarily as a reference for adjusting stimulation intensity in various TMS protocols. It serves to evaluate the corticospinal pathway's integrity and acts as a cortical excitability measure for individuals [17].

MT calculations can be made with the target muscle at rest (Resting Motor Threshold - RMT) or during slight tonic contraction (Active Motor Threshold - AMT). There are two principal approaches to determining MT:

1. *Relative Frequency Methods:* These methods systematically vary stimulus intensity, starting from lower values and incrementally increasing in a ramp-like manner. The aim is to establish a condition where MEPs are consistently evoked in 50% of attempts (10 out of 20) with amplitudes of at least 50 µV, for RMT, or 100 µV, for AMT, when the target muscle is in sustained contraction.
2. *Adaptive Methods:* These approaches employ a probability function to characterize the relationship between stimulus intensity and MEP occurrence. Each repetition involves recalculating the likelihood of evoking a MEP at specific intensity levels through an iterative process. The MT is identified as the intensity where there's a 50% probability of MEP occurrence [16].

## Input/output curve

MEPs generated by stimuli near the motor threshold typically have minimum amplitude. As stimulus intensity increases, so does MEP amplitude, up to a point where further intensity increases do not yield higher MEP amplitudes. The MEP amplitude's relationship to the stimulus intensity follows a sigmoidal pattern, known as the stimulus-response, recruitment, or input/output curve. This curve can be generated by fitting intensity and amplitude value pairs to a sigmoidal function, such as Boltzmann's equation.

From the input/output curve, researchers can extract three crucial parameters [18,19]:

1. *Slope (m):* The slope of the curve's linear-like portion, reflecting corticospinal excitability.
2. *MEPmax:* The maximum or saturation value of the MEP amplitude.
3. *S_50_:* The stimulus intensity required for the MEP to reach half of its maximum amplitude.

These parameters offer valuable insights into the state and responsiveness of the motor cortex under various conditions and interventions.

### Paired pulse

Paired pulse stimulation involves administering two TMS pulses to the same site with a variable interstimulus interval (ISI), allowing for the evaluation of phenomena like MEP attenuation or facilitation. The initial pulse is the conditioning stimulus, followed by the test stimulus. Adjusting the intensity of both stimuli and the ISI enables the assessment of various inhibitory and facilitatory corticocortical circuits.

Inhibitory phenomena observed with this technique are categorized into:

1. *Short-Interval Intracortical Inhibition (SICI):* A form of inhibition occurring over short intervals.
2. *Long-Interval Intracortical Inhibition (LICI):* Inhibition observed over longer intervals.

Facilitatory phenomena include [16]:

1. *Intracortical Facilitation (ICF): An increase in corticocortical excitability.*
2. *Short-Interval Intracortical Facilitation (SICF): Facilitation happening over short intervals.*

These phenomena offer valuable insights into cortical circuit excitability and connectivity.

Conducting a paired pulse protocol necessitates a specialized TMS machine capable of delivering two stimuli with differing intensities and an ISI as brief as 1 ms. This equipment enables researchers to explore and understand the nuanced interactions and responses within cortical circuits effectively.

### Motor evoked potential (MEP) amplitude

In evaluating motor cortex excitability changes post-intervention, whether pharmacological or via neuromodulation (rTMS), assessing the mean amplitude of MEPs provides an efficient, straightforward approach. While the input/output curve (I/O curve) offers valuable insights, acquiring it is time-consuming, potentially causing the excitability effects to dissipate or be influenced during the process.

Typical protocols under this paradigm involve an initial baseline measurement pre-intervention, followed by a post-intervention measurement. However, there is variability across studies in the number of baseline and post-intervention recordings obtained, and the stimulus intensity applied to the motor cortex. Standardization of these parameters is crucial for ensuring comparability between studies. Most protocols utilize a fixed stimulus intensity for all participants, often ranging from 120% to 140% of the resting motor threshold (RMT), or the intensity needed to evoke 1 mV amplitude MEPs [20], [21], [22], [23], [24], [25].

Yet, MEP amplitudes significantly vary among individuals, especially those with pathologies. For some, the intensity required to elicit a 1 mV MEP may be 120% of their RMT, while for others, this intensity may generate their maximum MEP amplitude. Given that individuals exhibit different slope magnitudes on their I/O curve, a fixed value like 130% of the RMT might position one person's MEP amplitude at the start of their I/O curve's rising portion, while for another, it might be near the plateau region.

For accurate assessment post-intervention, the chosen intensity should be where MEPs have equal likelihoods of increasing or decreasing in amplitude, corresponding to the *S_50_* of the I/O curve [16]. Thus, the method outlined here measures changes in cortical excitability using MEP amplitude, with an intensity equal to the individual's I/O curve's *S_50_*.

### Factors affecting MEP amplitude

MEP amplitude is subject to influence by a combination of physical and physiological factors [17]:

Physical factors:

• *Pulse type:* The kind of pulse used for stimulation, whether monophasic or biphasic, affects MEP amplitude.
• *Pulse duration and waveform:* The duration and waveform of the pulse also play crucial roles.
• *Coil characteristics:* The stimulation coil's shape, size, and orientation relative to the sagittal plane impact the MEP amplitude.

Physiological factors:

• *Coil-cortex distance:* The effective distance between the coil and the cerebral cortex, primarily determined by the thickness of the skull over the stimulated area, influences MEP amplitude.
• *Brain anatomical variations:* Differences in gyral orientation and white matter anisotropy, as well as corticospinal fluid distribution within the skull, also play roles.
• *Biological rhythms and states:* The circadian cycle, alertness or sleepiness levels, hormonal levels, and menstrual cycle stages can all affect MEP amplitudes.

Understanding and considering these factors are vital for interpreting MEP amplitudes accurately and consistently across different individuals and contexts. Each of these elements can contribute to the variability seen in MEP measurements, making it essential to control or account for them in experimental designs and data analysis.

### Materials

This detailed section provides an exhaustive description of all necessary equipment and materials required to successfully replicate the method described, ensuring utmost clarity and precision for the sake of reproducibility.

#### Patient setup materials

• Recording room: secure a recording space that is not only devoid of noise and visual distractions but also spacious enough (at least 6 m2) to comfortably accommodate the required equipment and facilitate easy maneuvering during the process.
• Chair selection: employ a specialized TMS chair or a comfortable, adjustable armchair. The ideal chair should offer adjustable features including seat rotation, vertical movement, and back tilt. If the chair comes with a headrest, it should be height-adjustable to prevent any interference with the accurate positioning of the TMS coil.
• Disposable surface electrodes: opt for Ag/AgCl electrodes, which are crucial for the recording process.
• Alcohol swabs: these are essential for sanitation and preparation purposes.
• TMS device: a transcranial magnetic stimulator complete with a figure-of-eight coil is imperative. For reference, our group has utilized the Magstim Rapid2, which comes with a 70 mm air-cooled figure-of-eight coil, manufactured by The MAGSTIM Company LTD.
• Biosignal amplifier: an amplifier or electromyograph equipped with a digitizer is needed. Ensure it comes with two bipolar channels, an adjustable range of amplification, a minimum resolution of 8 bits, and a sampling rate of no less than 10 kHz. Adjustable filters are a preferable feature. In our work, we have successfully used the MepPod, manufactured by The MAGSTIM Company LTD.
• Additional accessories (optional but recommended): consider having on hand a soft pillow, a thin cap or EEG cap for patient comfort, conductive EEG/ECG gel, and a soft sponge ball (approximately 7 cm in diameter).

#### Neuronavigation setup materials

• *Neuronavigation system:* a system that typically includes a tracking device or camera, toolkit with markers, and accompanying software is mandatory. We have experience using the Visor2 neuronavigation system manufactured by ANT Neuro. This system integrates an NDI Polaris Vicra camera from Northern Digital Inc. and Visor2 software from Eemagine Medical Imaging Solutions GmbH.
• *Tracking and calibration tools:* You'll need tracking tools (for the coil and patient's head) and calibration tools essential for the process.
• *Optional accessories:* eyeglass frames can be used to secure the tracking tool to the patient's head securely and comfortably.

#### Hotspot identification materials

• *Support arm:* a robust support arm capable of holding and manipulating the weight of the TMS coil is non-negotiable. We have previously used the Manfrotto Magic Arm Kit, complemented by a Super Clamp and a custom wheelbase for enhanced stability and adjustability.

#### Resting motor threshold acquisition materials

• *Android device: an Android-operated device, either a smartphone or tablet, is necessary. Ensure it is compatible with and can effectively run the ATH-tool app [* *26* *].*

#### Input/output curve and S50 calculation materials

• *Software requirement:* for data calculation and analysis, employ software or a statistical package that supports custom equation curve fitting. We have had success using MATLAB software (equipped with the Curve Fitting Toolbox) and Python compilers installed with essential libraries like NumPy and SciPy for seamless data processing and analysis.

Providing a meticulous list of materials with specifications and optional accessories ensures that other researchers can replicate your setup with high fidelity, promoting the reproducibility and reliability of your findings in the broader scientific community. Ensure you adhere to each specification for optimal results.

### Methods

#### Patient setup

##### Introduction to TMS

• For patients unfamiliar with TMS, provide an overview of the process, underscoring its non-invasive, generally painless nature.
• Highlight that the muscle may twitch involuntarily, and some discomfort may be felt due to facial muscle or nerve stimulation.

#### Seating arrangement

Seat the patient on an armchair comfortably. The patient should stay relaxed and still; hence, ensure comfort as the procedure might last for some minutes.
Consider using a lumbar support pillow for prolonged sitting comfort. Optionally, place a thin cap on the patient's head for hygiene and later assistance in locating the primary motor cortex area.

#### Target muscle identification

• Select the target muscle, with the abductor *pollicis brevis* (APB), *first dorsal interosseous* (FDI), and *abductor digiti minimi* (ADM) being commonly used due to their easy identification [2].
• The choice of muscle depends on the study's objectives. Although this procedure can be applied to various muscles, the primary focus here is on hand muscles.

#### Skin cleaning

• Clean the skin area where electrodes will be placed using alcohol swabs. This process is crucial for reducing electrode-skin interface impedance and ensuring effective electrical contact.

#### Electrode placement

• Employ surface Ag/AgCl electrodes on the target muscle in a belly-tendon arrangement. For the FDI muscle, as an example, place one electrode over the muscle belly (identified by having the patient press the index finger against the thumb) and connect this to the positive input of the amplifier.
• Place a second electrode over the muscle's insertion region and connect it to the amplifier's negative input. The third electrode should be placed on the ulnar styloid process, serving as a ground.

#### Patient guidance

• Instruct the patient to stay awake, alert, and relaxed throughout the procedure. They should keep muscles relaxed and avoid significant head movements to guarantee accurate data collection.

#### Equipment configuration

• Activate the TMS and EMG devices, adjusting necessary parameters. For devices like the Magstim Rapid^2^, you should select the single pulse mode, set the stimulator's output power, and configure the EMG amplifier's acquisition parameters accordingly.
• Recommended settings for the Magstim Rapid^2^ include an acquisition time of 100 ms (minimum), a bandpass filter ranging from 20 Hz to 10 kHz, and a scale of 100 µV per division.

#### Initial test pulses

• Administer a few TMS pulses into open air for functional testing and to acquaint the patient with the stimulation coil's sound. Concurrently, ensure that the EMG signal is free from electrical or muscle noise. Address any noise by recleaning the area or replacing the electrode gel.

#### Muscle relaxation verification

• During periods of muscle relaxation, the EMG signal should be steady with an amplitude near zero. If not, assist the patient in finding a comfortable, relaxing hand position. If relaxation is still challenging, engage the patient in a repetitive squeezing and releasing exercise using a small sponge ball to facilitate muscle relaxation.

## Neuronavigation setup

Neuronavigation is integral in pinpointing the TMS coil's precise location relative to the designated brain stimulation area, ensuring consistent and reproducible stimulation across various sessions and operator [27]. These devices range widely, from systems employing radio frequency to locate landmarks on the skull to contemporary optical tracking systems. The latter utilizes either active (light-emitting) or passive (reflective) markers, which can be spatially tracked using one or more cameras [28].

In this section, the neuronavigation procedure is outlined based on the ANT Neuro neuronavigation system (please refer to the Materials section for additional details). Nevertheless, the steps provided herein are generally applicable to most neuronavigation systems and aim to offer a broad description suitable for diverse equipment and applications.

### Calibration procedures

#### TMS equipment and neuronavigation system calibration

• Placement of markers, either active or passive, is essential on both the stimulation coil and the patient's head.
• The calibration tools utilize these markers to determine the distance between the coil markers and the point of the highest magnetic flux density, where maximal stimulation occurs.
• Engage in this calibration process at the beginning of each TMS session to ensure accurate results.

#### Neuronavigation system calibration with anatomical data

• Modern systems can load and utilize patient-specific MRI or CT data to generate a 3D brain model. Systems may also have preloaded standard brain models for use.
• These models assist in identifying the specific cortical area targeted for stimulation and calculating the electric field in that tissue area.
• A crucial step involves the calibration procedure that matches the 3D model to the patient's head. This involves marking anatomical reference points on the patient's head, which the system will detect. These markers can be secured using adhesive markers or an elastic band.
• For systems like ANT Neuro, use a pointer tool to mark specific anatomical points, such as the nasion and the preauricular points. Additionally, recording spatial points on the patient's skull surface is necessary to adjust the 3D model to the patient's head size accurately.

### Stability of markers

• Marker stability is crucial. Any inadvertent movement can lead to the decalibration of the neuronavigation system, leading to inaccuracies in the stimulation point.
• One method to ensure marker stability is to place them on eyeglass frames, secured further with an elastic band. This approach minimizes the risk of displacement. If the markers do move, they can be repositioned without necessitating a recalibration, provided the glasses were well-seated during the initial setup.

### Defining target points

• If the patient's imaging study is loaded into the neuronavigation software, you can anatomically mark the point corresponding to the primary motor cortex on the 3D model. This precise marking allows for the initiation of direct stimulation to the specified area.

### Notes for various systems

• The outlined procedure is based on the ANT Neuro neuronavigation system but is generally applicable to most neuronavigation systems available.
• Though different commercial devices might utilize various techniques, ranging from radio frequency systems that locate skull landmarks to advanced optical tracking systems with active or passive markers, the general principles of setup and calibration are similar.
• Always refer to the specific user manual and guidelines provided by the equipment manufacturer for detailed instructions and considerations pertinent to the particular system you are using, ensuring accurate and reproducible results in your TMS studies.

## Finding the hotspot

Identifying the hotspot, or the optimal stimulation point on the primary motor cortex, is vital as it is the coil position that maximally induces motor-evoked potentials (MEPs) in the target muscle. Methods for locating this pivotal point are varied, including manual search, MRI-based localization, amplitude mapping, and automated searches facilitated by robots [29,30].

A manual search offers a practical, accessible approach, necessitating minimal equipment, materials, and time, while retaining sufficient accuracy. This method is particularly advantageous when there is no availability of patient MRI data or when robot-assisted TMS resources are limited. Below are the meticulous steps for a manual hotspot search:

### Preparation

• Ensure all devices, including TMS equipment, EMG, and neuronavigation systems, are activated and configured correctly for the process.

### Coil positioning

• Gently position the stimulation coil on the patient's scalp without exerting undue pressure. The coil's point of maximum magnetic flux density should align meticulously with the primary motor cortex area (M1).

### Identifying M1

• The maximum intensity point within the coil varies depending on its design. For a figure-of-eight coil, this point is centrally located at the line connecting the centers of the two loops. You can identify the M1 on the precentral gyrus, recognizable by its omega-shaped structure [31]. With an EEG cap marked per the 10-20 system, M1 resides between positions C1-C3 or C2-C4 on the left and right hemispheres, respectively [32].

### Coil orientation

• Set the coil at a 45° angle relative to the sagittal plane, ensuring the induced current flows perpendicularly to the central sulcus for optimal stimulation [16,33].

### Intensity calibration

• Initiate stimulation at approximately 35% of the TMS device's maximum power output. Incrementally elevate the intensity in 5% steps, administering stimuli until MEPs with an amplitude around 250 µV are consistently generated.

### Stimulation and marking

• Mark the initial stimulation spot on the neuronavigation system. Move the coil frontally about 10 to 15 mm from the initial point, maintaining its orientation, and administer 3 to 4 more stimuli with a minimum of 5-second intervals.

### Iterative testing

• Repeat the above step, repositioning the coil dorsally, medially, and laterally relative to the initial point and apply the designated number of stimuli at each new location. If an alternate location yields a higher mean MEP amplitude, mark this as a potential hotspot and repeat the process until the definitive hotspot is identified.

### Finalizing hotspot identification

• The definitive hotspot is the location where MEPs consistently exhibit the highest amplitude or most stable response at a constant intensity.

### Documentation

• Upon hotspot identification, diligently record its coordinates or save the point in the neuronavigation software for reference. Additionally, utilizing a support arm to maintain the coil's position over the hotspot throughout the session is highly recommended for consistent, accurate results.

## Obtain resting motor threshold

Various techniques exist for calculating the Resting Motor Threshold (RMT), each with different advantages. The adaptive methods stand out due to their speed, accuracy, and requirement of fewer stimuli, making them particularly efficient in the RMT estimation process.

A noteworthy tool facilitating this process is the ATH-tool application, developed by Petro Julkunen, which is freely available for Android devices. This application utilizes the Parameter Estimation by Sequential Testing (PEST) algorithm to compute the RMT, typically requiring only 14 trials in most cases for an accurate estimation [26]. The procedure for using this tool is outlined below:

### Introduction to RMT calculation methods

• Calculating the Resting Motor Threshold (RMT) is essential for TMS studies. Among the various methods available, adaptive techniques are prominent due to their expeditious and accurate calculations, necessitating fewer stimuli overall.

### Utilizing the ATH-tool application

• The ATH-tool application, a free and invaluable resource developed by Petro Julkunen, is designed for Android devices. This application incorporates the Parameter Estimation by Sequential Testing (PEST) algorithm, usually requiring only around 14 trials to accurately estimate the RMT.

#### Pre-procedure preparations

• *Subject orientation:* Instruct the subject to maintain a state of relaxation, keeping their hands in a stable and comfortable position. Movement should be minimized to ensure the accuracy of the measurements taken during the procedure.

#### Application set-up

• *Application launch:* initiate the ATH-tool application on your Android device.
• *Initial Threshold Guess:* If MEPs begin to appear at a specific stimulation intensity during the hotspot identification phase, use this value as the initial RMT guess within the application. Select the “Threshold with Good Guess” option. If there's no initial guess, select the “Threshold Between 20 and 80” option.

#### Stimulation process

• *Stimulation initiation:* upon pressing the application's “Start” button, adjust the TMS device's intensity to correspond with the app-suggested value for the next testing phase. Place the coil over the hotspot and administer a stimulus at this intensity.
• *Response logging:* if a stimulus elicits an MEP of at least 50 µV in amplitude, press the application's “Response” button (identified by a thumbs-up icon). In the absence of a MEP, press the “No Response” button (represented by a thumbs-down icon).
• *Intensity adjustment:* the application will subsequently suggest a new intensity level for the next stimulus. Adjust the TMS device to this new intensity level and administer another stimulus, maintaining a pause of at least 5 seconds between each stimulus to ensure accurate data collection.

#### Completion of RMT calculation

• *Concluding the Procedure:* Continue following the application's prompts and administering stimuli until a green dove icon appears on the upper right-hand corner of the application interface. This icon signals that the RMT has been successfully calculated, aligning with the set confidence levels for accuracy and reliability.

### Post-procedure considerations

• Upon completion of the RMT calculation process using the ATH-tool application, it is imperative to carefully document the obtained values. These values are crucial for customizing the intensity levels in subsequent TMS protocols and ensuring consistency and reliability in the data collected throughout the study.

Detailed adherence to each of these enumerated steps is vital to accurately obtain the RMT, a pivotal metric in TMS studies. Following this comprehensive methodology ensures that the process is replicable, fostering consistency and reliability in TMS research across various settings and studies.

## Input/output curve and S_50_ calculation

Following the determination of the Resting Motor Threshold (RMT), you will need to generate an input/output (I/O) curve, which facilitates the calculation of the intensity corresponding to *S_50_*. This intensity is essential for subsequent stages of the procedure. Several methodologies for creating this curve are documented in existing literature, each varying in terms of stimulus intensity variation (incremental, decremental, or random), the count of recorded Motor-Evoked Potentials (MEPs) per level of stimulation, and the interstimulus interval [34,35].

It's important to note that specific methodologies, particularly those involving variations in stimulus intensity, may induce a hysteresis effect during the curve measurement process. This effect is especially prominent when dealing with short interstimulus intervals [36]. To circumvent this issue, one could opt for randomly selecting intensity levels. However, this approach introduces a degree of complexity to the procedure, which is further exacerbated if the Transcranial Magnetic Stimulation (TMS) device in use lacks a programmed sequence for random intensity variation.

For the purposes of this method, an incremental approach to stimulus intensity variation has been chosen for its simplicity. Since the primary objective here isn't to draw comparisons between different I/O curves, the potential hysteresis effect observed with this approach does not critically affect the calculation of the *S_50_* parameter.

Outlined below are the steps to generate the I/O curve:

*1. Adjusting TMS output:* upon determining the RMT, while keeping the coil positioned over the hotspot, adjust the TMS output to match the RMT value.

*2. Initiating stimulation:* launch the stimulation process with a frequency set at one pulse every six seconds. With the intensity set at 100% of the RMT, it's anticipated that approximately half of the stimuli will elicit MEPs with a minimum amplitude of 50 µV. It's advisable to keep track of the count of stimuli at 100% RMT that successfully induce an MEP. Should the ratio of applied stimuli to generated MEPs diverge significantly from 50 %, consider recalculating the RMT, as detailed in step 4.

*3. Recording MEPs:* continue the stimulation until you have recorded a total of 10 MEPs. Following this, increase the TMS output intensity by 10% (equivalent to 110% of the RMT) and proceed to record an additional 10 MEPs.

*4. Incrementally increasing intensity:* persist with this process, incrementally increasing the stimulus intensity in steps of 10% of the RMT for every 10 MEPs recorded. Continue until you reach 180% of the RMT or hit the maximum power output limit of the TMS device.

*5. Data acquisition and analysis:* upon completing the acquisition of the I/O curve, calculate the average amplitude of the 10 MEPs associated with each intensity level. This will yield a series of ordered pairs, each consisting of an intensity level (ranging from 100% to 180% of the RMT) and its corresponding average MEP amplitude.

*6. Data fitting with software:* employ calculation software or a statistical package to fit the acquired data to Boltzmann's sigmoidal equation [18]. Ensure you have the necessary computation tools and statistical packages installed and ready for this step of the process:

$$MEP\left(s\right)=\;\frac{MEP_{max}}{1+e^{m\left(S_{50}-s\right)}}$$

In the sigmoidal equation, various fitting parameters are represented: *m* denotes the slope, *MEP_max_* ​ indicates the maximum value of the MEPs amplitude, and *S_50_* corresponds to the stimulus intensity that elicits a MEP equating to 50% of *MEP_max_*. For illustrative purposes, we provide example codes for performing this fitting using both the MATLAB numerical computing platform and the Python programming language, delineated in Table 1 and Table 2, respectively.

**Table 1:** MATLAB script: This script is designed to fit the parameters of a Boltzmann sigmoid equation to the MEPs amplitude data and to calculate their 95% confidence intervals. Be aware that execution of this script necessitates the MATLAB Curve Fitting Toolbox.

**Input**	***x***: vector containing the intensity values of the stimuli. ***MEP***: vector containing the average of the amplitudes of the MEPs at each stimulation intensity.
**Output**	***IOCurveFit***: object containing the fitting parameters. Fitted parameters values can be accessed individually by typing *IOCurveFit.MEPmax, IOCurveFit. S_50_* and *IOCurveFit.m*.

**Script example**	fitOpts = fitoptions('Method','NonlinearLeastSquares','StartPoint',[5 140 1], ... 'Lower',[0,0,0],'Upper',[25,180,100]); fitType = fittype('MEPmax/(1+exp(m*(*S_50_*-x)))','options',fitOpts); x = [100 110 120 130 140 150 160 170 180]; MEP = [0.1821 0.1769 0.4022 0.8966 1.5531 3.0366 2.6929 2.9976 2.8393]; IOCurveFit = fit(x',MEP',fitType)

**Table 2:** Python code: the Python code listed in this table facilitates the acquisition of fitting parameters *MEPmax, S_50_* and *m*, along with their respective 95% confidence intervals. Ensure the NumPy library is installed to execute this script successfully.

**Input**	***x***: vector containing the intensity values of the stimuli. ***MEP***: vector containing the average of the amplitudes of the MEPs at each stimulation intensity.
**Output**	The *MEPmax, S_50_* and *m* parameters will be printed on the console (with the 95 %CI in brackets).

**Code example**	import numpy as np from scipy.optimize import curve_fit from scipy.stats.distributions import t x = np.array([100, 110, 120, 130, 140, 150, 160, 170, 180]) y = np.array([0.1821, 0.1769, 0.4022, 0.8966, 1.5531, 3.0366, 2.6929, 2.9976, 2.8393]) def MEP(s, MEPmax, *S_50_*, m): return MEPmax/(1+np.exp(m*(*S_50_*-s))) param, param_cov = curve_fit(MEP, x, y, p0=[10., 140., 0.1], bounds=(0, [25., 180., 1])) alpha = 0.05 # 95% confidence interval n = len(y) p = len(param) dof = max(0, n - p) tval = t.ppf(1.0-alpha/2., dof) sigma = np.sqrt(np.diagonal(param_cov)) print(“MEPmax = ”, param[0], “[”, param[0]-tval*sigma[0], “, ”, param[0]+tval*sigma[0], “]”) print(“*S_50_*= ”, param[1], “[”, param[1]-tval*sigma[1], “, ”, param[1]+tval*sigma[1], “]”) print(“m = ”, param[2], “[”, param[2]-tval*sigma[2], “, ”, param[2]+tval*sigma[2], “]”)

## Basal measurement

Upon obtaining the *S_50_* intensity value, proceed to the basal measurement stage. The basal measurement involves recording the amplitude of MEPs to estimate cortical excitability prior to the intervention.

### Repositioning the TMS coil

• Reposition the Transcranial Magnetic Stimulation (TMS) coil over the hotspot, utilizing the neuronavigation system. Ensure the coil's orientation remains consistent with previous positioning.

### Adjusting the TMS output power

• Modify the TMS output power to align with the previously obtained S50 value.

### Stimulation initiation

• Commence the stimulation of the hotspot, delivering a pulse at six-second intervals. Concurrently, record the elicited potentials within the EMG system. Continue this procedure until you've recorded a minimum of 30 MEPs. For a robust and reliable estimation of basal excitability, without unduly extending the session duration, we recommend capturing a total of 60 MEPs.

### Post-basal measurement procedure

• Following the completion of the basal measurement, you may initiate the planned intervention aimed at altering neuronal excitability. Interventions may include rTMS, pharmacological approaches, or neurorehabilitation procedures.

### Post-intervention measurement

The procedures for post-intervention measurement vary depending on the type of intervention implemented:

*Repositioning and Recalibration:*

• If electrodes and neuronavigation markers were removed during the intervention, they must be repositioned. The neuronavigation system also requires recalibration. Please refer back to Steps 1 and 2 for guidance on this process.

### Considerations for rTMS intervention

• For interventions involving repetitive Transcranial Magnetic Stimulation (rTMS), it's recommended to administer it immediately following the baseline measurement. After the rTMS application, promptly commence the post-intervention measurement to effectively capture the initial effects of the intervention.

### Procedure for post-intervention measurement

• Execute the post-intervention measurement similarly to the baseline measurement. However, the number of MEPs recorded will vary based on the duration of the affects you intend to measure.
• For example, in scenarios involving an rTMS protocol, capturing around 150 MEPs is advisable. This quantity corresponds to approximately 15 minutes of after-effects, assuming an interstimulus interval of 6 seconds. Ideally, the number of MEPs acquired should be equal to or exceed the number recorded during the baseline measurement for consistency and reliability in the data collected.

## Data analysis

Once a series of MEPs both pre- and post-intervention are obtained, it's essential to proceed with analyzing changes in cortical excitability as reflected by alterations in MEP amplitudes. This analysis can be achieved through various methods:

### MEP amplitude measurement

• Manual Measurement: This involves identifying and measuring the deflection in the EMG, from the negative trough to the positive peak, marking the beginning and end of each MEP.
• Automated Measurement: Alternatively, you can use a computer algorithm for automatic detection and measurement of MEP amplitudes [37].

### Addressing MEP amplitude variability

• Given the intrinsic variability in MEP amplitudes, even within the same subject and at identical stimulation intensities, averaging the amplitudes of multiple MEPs is critical for obtaining a stable and reliable measurement. Several studies have investigated the minimum number of MEPs necessary for a reliable assessment of corticospinal excitability based on amplitude. The general consensus recommends a range of 20 to 30 MEPs, with 30 MEPs providing an excellent estimate of the mean amplitude value [38], [39], [40], [41], [42].

### Baseline and post-intervention measurements

• A minimum of 30 MEPs is suggested for baseline measurements to ensure the derived amplitude value is representative. If more MEPs are obtained, consider grouping them into sets of 30 and calculating the average amplitude for each set. This methodology applies to both baseline and post-intervention measurements, facilitating a comprehensive analysis of changes over time.

### Calculating and comparing amplitude changes

• Amplitude changes between baseline and post-intervention stages can be represented as either the difference or the ratio of the average amplitudes. Employing a moving average with a 30-data-point overlapping window can offer valuable insights into the trend of MEP amplitude changes over time.

### Normalization for cross-subject comparison

**Fig. 1:**
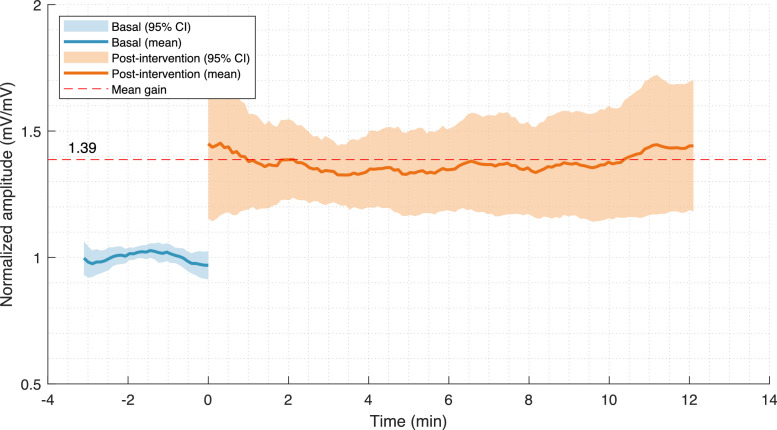
Comparative amplitude changes in MEPs following facilitatory rTMS intervention: The illustration shows the variations in MEP amplitudes following facilitatory rTMS intervention with a sample of 10 subjects. The baseline measurement is depicted by the blue graph (60 MEPs), contrasted with the orange graph which represents post-intervention measurements (150 MEPs). A moving average with a 30-sample window is applied to both datasets for smoother data interpretation. Data normalization was based on the mean amplitude derived from the baseline MEPs (60 in total). The shaded areas delineate the 95% confidence intervals, with the dotted line highlighting the observed amplitude gain throughout the post-intervention phase.

• For comparisons across multiple subjects, normalization of amplitude data is imperative. Achieve this by dividing each amplitude value by the average amplitude obtained during the baseline measurement, as depicted in Fig. 1. This process ensures that the data is comparable and interpretable across different subjects with varying baseline cortical excitabilities.

## Ethics statements

The data represented in Fig. 1 were sourced from a study that was diligently conducted with healthy volunteers, adhering strictly to the globally accepted ethical standards and recommendations outlined in the WMA Declaration of Helsinki. This declaration, entitled “Ethical Principles for Medical Research Involving Human Subjects”, alongside the Recommendations for the Conduct, Reporting, Editing, and Publication of Scholarly Work in Medical Journals, served as the guiding framework for the study's design, execution, and reporting. The study was conducted under the approved protocol (49-54-2018) sanctioned by Hospital General Dr. Manuel Gea González, located in Mexico City, Mexico. Furthermore, to ensure ethical integrity and transparency, written informed consent was duly obtained from every participant involved in the study.

## CRediT author statement

**Emmanuel Ortega-Robles** contributed to the conceptualization, methodology, software development, original draft preparation, and data curation. **Jessica Cantillo-Negrete** participated in visualization, investigation, and writing, specifically in the review and editing phases. **Ruben I. Carino-Escobar** was involved in visualization, investigation, and contributed to writing through review and editing. **Oscar Arias-Carrión** played a significant role in conceptualization, provided resources, engaged in review and editing of writing, and took on supervision and project administration responsibilities.

## Declaration of Competing Interest

The authors declare that they have no known competing financial interests or personal relationships that could have appeared to influence the work reported in this paper.

## Data Availability

Data will be made available on request.
